# Literature review on engineering surface modeling

**DOI:** 10.1016/j.mex.2023.102122

**Published:** 2023-03-11

**Authors:** Junye Ma, Lin Li

**Affiliations:** a School of Applied Sciences, Taiyuan University of science and technology, Taiyuan 030024, China; b Institute of intelligent manufacturing and control technology, Taiyuan University of science and technology, Taiyuan 030024, China

**Keywords:** Rough surface, Rough surface characterization, Numerical reconstruction, Contact analysis, Literature review, Literature review

## Abstract

This paper provides a comprehensive literature review of numerical simulation models for engineering surfaces. This review attempts to make a link between the development of mainstream models, especially focusing on recent advances in surface topography modeling research within the last 2-3 years, and presenting future perspectives on these aspects. However, it is important to note that surface topography modeling serves more of a subsequent tribological simulation. Tribology is a highly multidisciplinary field, and its research results are widely distributed across various disciplines. Therefore, some literature may have been missed in this review. This review attempts to make a link between the development of mainstream models, and presenting future perspectives on these aspects. We hope that such a review will lead to the latest focused research and be useful to the growing community of theoretical research on engineering surface modeling.

## Review Highlights

• The history of the development of the major models is sorted out.
• The papers from the last two years are in focus.
• The future directions of development are prospected.

## Graphical abstract

**Figure:**
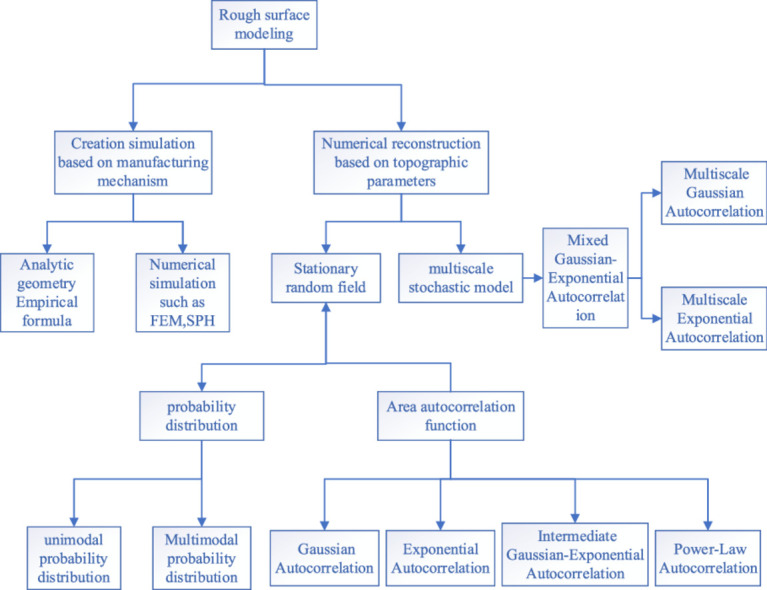

Specifications table

**Table:** 

Subject area	Engineering
More specific subject area:	Surface analysis, Surface integrity
Name of the reviewed methodology:	Literature review
Keywords:	Rough surface, Numerical reconstruction, Literature review
Resource availability:	none
Name and reference of original method:	L. Li, J. Tang, H. Ding, D. Liao, D. Lei, On the linear transform technique for generating rough surfaces, Tribology International 163 (2021) 107182.
Review question:	Review of rough surface modeling

**Method details**

## Overview

With the development of smart manufacturing, the digital prediction and optimization of part performance is receiving more and more attention from industry. The surface with machined topography features, as the input for the digital prediction of topographic properties, its parametric characterization and computer numerical generation technology are the guarantee for the effective implementation of smart manufacturing.

### Rough surface characterization

A prerequisite for numerical modeling of rough surfaces is the validity of the representation. Surface topography parameters are the most important means of characterization.

There are currently two dominant categories of ideas in surface characterization, one of which is the classical random field model introduced by Longuet-Higgins [1]. Based on the stationary random field theory, most of these methods characterize the topography with height distribution parameters and spatial distribution parameters, the relevant description of the parameters is described in detail in Ref. [2]. When analyzing contact problems, parameters such as peak density and radius of curvature are used accordingly, mainly represented by GW-like statistical contact models. Nayak performed a systematic analysis of the random field model and gave a theoretical formulation for the direct calculation of the GW model topography parameters by the spectral distance method [3]. Later, Nayak analyzed the plastic contact problem of Gaussian isotropic rough surfaces [4]. However, as Greanwood and Wu pointed out, there are some minor flaws in the peak density determination problem of the GW model [5]; various improved semi- analytic models have been developed in the last decade. The other type of analysis is carried out from the multiscale aspect, based on the cross-scale properties of the surface. The fractal model introduced by Gagnepain and Roques-Carmes [6] is the leading idea, which was used by Majumdar [7] to analyze the rough surface contact problem to solve the problem of the variation of topographic parameters with the measurement scale. Of course, there are other multi-scale models, including wavelets, and superposition models of simple microconvex bodies.

The autocorrelation function contains important spatial information for the characterization parameters of rough surfaces. Peklenik measured the surface topography of various types of processing and classified the form of autocorrelation functions into five categories [8]. Loukjanov pointed out that the autocorrelation functions of engineered surfaces can be divided into two categories, one that decays rapidly and then oscillates around zero, and the other that oscillates continuously during a slow decrease [9]. Nayak established the relationship between each isotropic surface profile autocorrelation function and topographic autocorrelation function [10]. Considering that the surface texture of modern processing is more obvious, the traditional autocorrelation function is decomposed into random and deterministic components to characterize this phenomenon [11]. Similarly, the idea of ridge waves [12] is used to characterize spatial features with the help of graphic images, and texture segmentation is used to distinguish the texture structure of different factors [13].

These two-dimensional signal processing models applied to three-dimensional rough surface modeling theory have greatly advanced the rough surface characterization system and are of great significance for dealing with complex textures. The most sharp problem in characterization at present is that the connection and distinction between different characterization systems are not well defined. For example, even for the same surface contact problem, scholars with a physical perspective may prefer a fractal characterization system, while scholars with a machining perspective are more likely to adopt a random field characterization system. In addition, even for scholars who belong to the manufacturing field, there is still no well established characterization system for different manufacturing methods, such as grinding, turning, and shot peening processes.

### Rough surface reconstruction

In fact, as early as 1978, Patir [14] realized the value of numerical generation techniques for surface roughness in one's analysis of the role of surface roughness in friction systems. He gave a computational framework for generating surface roughness with specified statistical properties based on the MA-2D model and applied it to the analysis of hybrid lubrication. The computational framework was so successful that it is still one of the dominant frameworks for generating surface roughness until today. The disadvantages of Patir's reconstruction model are mainly computational, firstly, in solving the sliding mean coefficient, because the system of equations is nonlinear and large, Newton's method is introduced to solve it, but the more essential problem is that the exact solution of this nonlinear system of equations may not exist; secondly, in generating the non-Gaussian surface roughness, because the probability density function of the random field obtained from the linear filter acting on the non-Gaussian random field is not only related to this filter kernel but also to the probability density of the original non-Gaussian random field, it is necessary to introduce an additional nonlinear system of equations to constrain this relationship.

To solve the first problem, i.e., to improve the efficiency of solving largescale nonlinear equations, Hu and Tonder [15] proposed the use of finite response impulse filtering to implement the reconstruction based on the linear filtering nature of the MA-2D model. This method achieves the calculation of filter coefficients in the frequency domain by the FFT method, which avoids the problem of solving nonlinear systems of equations and thus greatly improves the computational efficiency. However, Hu and Tonder ignore the fact that the FFT corresponds to a circular convolution rather than a linear convolution operation, and Wu [16] gives an alternative expression for the FFT method to generate a Gaussian surface based on the law that the Gaussian varies uniformly with the corresponding phase of the field. In addition, some scholars believe that the FFT model has shortcomings in dealing with problems of large correlation length, so they still work on optimizing the solution of the nonlinear system of equations in the original MA-2D model. Because the exact solution of the equations is not stable, this part of the work focuses on transforming the original equations into an optimization problem and proposing different optimization methods to compute the regular solutions of the equations. Bakolas [17] directly optimized the original equations and reduced the computational space complexity by introducing the nonlinear conjugate gradient method. In the literature [18], the validity of the method was verified by comparing the characterization parameters of the MA-2D model-generated morphology with those of the measured morphology in 3D. Liao [19] advocated the optimal calculation of the *F*_0_-norm of the original equation and gave the analytical gradient. Shortly, the error problem in the Liao's model is further improved by using a symmetric linear transformation matrix [20]. Finally, the linear filtered reconstruction problems for rough surfaces with arbitrary texture directions are combined into a unified model [21] that is very accurate but time-consuming to solve compared to the FFT method.

To address the second problem, i.e., non-Gaussian surface generation. Watson and Spedding [22] used the Johnson transformation system to simplify the process of generating non-Gaussian engineered surfaces in a linear filtering model by inferring the bias and peak states of the initial distribution from the bias and peak states of the target surface. The FFT method of Wu [23] takes advantage of the fact that phase changes do not affect the power spectral density when generating non-Gaussian random surfaces. Francisco pointed out the three problems in Hu's FFT model from the measured engineering surface measurement and reconstruction, and gave an analytical non-Gaussian random number generation method, and proposed for the first time to optimize the reconstruction effect by adjusting the height ordering [24]. Wang used a random exchange to adjust the spatial arrangement of the height distribution to achieve an approximation of the ideal autocorrelation function [25]. Wang [26] combined the FFT method with the theory of transport processes, and used the iterative transport approximation method to achieve the effect of the ideal autocorrelation function. However, these methods are limited to the problem of coupling the autocorrelation function with a high distribution. Eventually, this status was changed by the iterative adjustment method [27].These reconstruction methods are established for the stationary random field surface characterization theory of Longuet Higgins and Nayak. However, the rough surface is non-stationary, i.e., the variance is not unique with the sampling length. Moreover, the researchers found that the input parameters for the GW model to calculate the contact, such as the top density, the average radius of curvature of the top, etc., are not essential features of the surface, and they deviate to different degrees with the sampling spacing. Obviously, the reconstructed model for the height probability density functions, i.e., mean, variance, skewness, and kurtosis, does not reflect the multi-scale characteristics of the original surface. The introduction of fractal characterization parameters for surface morphology led to a change in the morphological reconstruction framework. Gagnepain and Roques-Carmes first proposed the use of fractal functions to characterize rough contours. Later, Majumdar and Bhushan started to use the Weierstrass-Mandelbrot (W-M) fractal function to reconstruct the surface profile of each homogeneous surface and to analyze the contact properties. The fractal structure was also used to analyze various physical properties such as wear and tear, and more satisfactory results were obtained. Then, Blackmore and Zhou [28] proposed a generalized W-M fractal function that can characterize most of the known physical properties and theories of engineering surfaces and build surface simulation models based on it. The method of fractal model based on Monte Carlo simulation is given by Zhou [29]. At this point, the fractal surface simulation has met the basic requirements. However, the characterization parameters based on the fractal model (fractal dimension) are not as easily understood physically as those based on the random field model (mean radius of curvature of the peak, etc.), and there is still no consensus in the academic community as to whether the surface of a metal with plastic flow processing has fractal characteristics. Therefore, Pérez-Ràfols and Almqvist [27] analyzed the stability in the case of exponential and fractal autocorrelation functions, respectively, when reconstructing the morphology based on the amplitude-adjusted Fourier transform method. Blackmore and Zhou established the relationship between the fractal parameters and the support rate curve. While Podsiadlo, Wolski and Stachowiak achieve each anisotropic fractal surface by controlling the curvature of the directional cover, and then establish the link between the radius of curvature and the fractal parameters. In addition, with the recent development of structural miniaturization, Motif has become increasingly popular among scholars to characterize the effect of structured surfaces on performance. Zahouani detailed the theoretical approach of morphological filtering and identification of each anisotropic component by spectral roses [30]. Sott pointed out that the Motif approach to characterize the morphology has a significant advantage over the traditional average-based parameters [31]. Bruzzone gave a method to analyze the evolution of steel surface texture structure with reciprocal sliding under hydrodynamic lubrication conditions [32]. Bruzzone gave surface topography parameters to characterize the friction properties [33]. In this context, ecological characterization methods were introduced to characterize the surface topography. On the one hand, ecological parameters are used to analyze the morphological surfaces of MEMS originals, such as morphological parameters (prefixed with the initial M of Morphological): Mff for measuring the anisotropy of the primitive from the area point of view, Mar for the ratio of primitive length to diameter, Mrn for the roundness of the primitive, Med for the equivalent diameter of the primitive, and so on, On the other hand, after the ecological filtering process, some traditional parameters were modified accordingly, such as significant peak density Spd, significant peak curvature Spc, significant On the other hand, after ecological filtering, some traditional parameters were modified accordingly, such as significant peak density Spd, significant peak curvature Spc, significant height S10z, etc. (By significant, we mean that the larger micro-convexity was selected after proper filtering). The Motif method was used to analyze the ecologically filtered The Motif method can effectively avoid the discussion of the sampling spacing problem [34]. From the contact point of view, this method is an excellent solution if the filtering scale is reasonably considered [35]. This method is an excellent solution if the filtering scale is considered reasonably. In a sense, ecological filtering is analogous to the abrasion process, in which small-scale morphological features are filtered out. However, it is based on morphological segmentation methods such as the watershed algorithm, which is more inclined to adaptive data analysis and does not have a good theoretical basis, and is therefore difficult to It is difficult to standardize and cannot be widely used in industry [36].

In fact, graph and network theories are the new way out in view of the complexity of surface morphology [37]. Wolf applied cohesive graph theory to morphological characterization and achieved good results in dealing with scale adaptation [38].

In addition, it is worth mentioning that morphological reconstruction has long been not limited to reconstructing surface roughness. Although Nayak proposed the reconstructed model for surface roughness, Watson and Spedding have explicitly pointed out the idea of using height and spatial features to reconstruct engineered surfaces. The idea of using height and spatial features to reconstruct engineered surfaces has been clearly stated by Watson and Spedding. Moreover, with the development of research on surface properties, friction, wear, lubrication, and many other properties are receiving more and more attention from the industry, surface Jablonski superimposed fractional components on random components [39] to reconstruct the engineered surfaces. Pawlus [40] gave a method to simulate the surface formed by two processes and after a “zero-wear” treatment [41]. He did this by combining the FFT reconstruction of the single-layer morphology by linear superposition to construct a surface that satisfies the specified parameter Rmq. Based on this, Hu further developed the Bi-Gaussian reconstruction method to reconstruct the wear surface, which combines the superposition method with probabilistic material support curves and is more advantageous in modeling the morphological properties and their evolution. The method combines the superposition method with probabilistic material support curves, which is more advantageous in modeling the morphological properties and their evolution. Recently, Hu [42] et al. pointed out the bi-fractal characteristics of surface roughness and combined the bi-Gaussian reconstruction method with fractal reconstruction to give a bi-fractal bi-Gaussian surface reconstruction method. On the other hand, some simulation methods based on machining mechanisms have been used to generate surfaces generated by the corresponding machining methods, such as pure abrasive cut workpiece models, and their improved models considering physical effects such as plowing, extrusion, etc. models to generate ultrasonic grinding surfaces.

In terms of reconstruction, the reconstruction based on specific characterization parameters can basically meet the parameter demand, and the reconstruction based on processing mechanism can basically meet the feature demand. However, limited by the problem of accurate characterization of specific processing textures, reconstruction based on specific representational parameters cannot solve certain processing textures (e.g., shot peening surfaces with large feature scales), which limits the progress of intelligent manufacturing toward the digital twin. Technologies that have emerged in recent years, such as deep learning-based image generation, are expected to make a breakthrough in this direction.Given the above background, the aim here is to provide a comprehensive literature review of parametric characterization and reconstruction aspects of engineering surfaces, which including parametric characterization as well as numerical reconstruction. While Section 2 critically analyses the theoretical framework of the engineering surface reconstruction. Section 3 reviews the representative papers of the last two years. Section 4 concludes the study.

## Methodology

Any reconstruction method has to target a set of topographic statistical parameters, and the specified topographic parameters are used as reconstruction targets. The classical random field reconstruction model considers that the topography can be completely determined by the height distribution and autocorrelation function, so the goal is to reconstruct the surface with the specified height distribution parameters and autocorrelation function.

### Parametric characterization of engineering surfaces

The surface topography of a component is intrinsically three-dimensional and therefore the physical behavior associated with the surface also occurs in three dimensions. For this reason, characterization and analysis of the surface form in three dimensions will be more effective in understanding the properties of the surface.

In the three-dimensional Euclidean coordinate system, the physical surface can be represented as a function *f* (*x, y*) of two independent variables x, y. However, in practical engineering, the surface is sampled by the measuring instrument on the function *f* (*x, y*), i.e. the height matrix

(1) $$Z=z\left(x_{i},y_{j}\right)\left\{ \begin{array}{cc} x_{i}\in X, & X=\left\{0,1,\cdots ,n_{x}-1\right\}\\ y_{j}\in Y, & \;Y=\left\{0,1,\cdots ,n_{y}-1\right\} \end{array}\right.$$

where $n_{x},n_{y}$are the number of pixels lattice in the x, y directions, respectively. In this way, we establish a correspondence between the surface topography and the height matrix Z through the sampling spacing. Although the sampling spacing (δx, δy) may be found to vary, the number of sampling lattice points ($n_{x},n_{y}$) corresponds directly to the size of the matrix, transforming the problem of characterizing the surface topography into one of characterizing the height matrix. Therefore, the surface interpolation problem is essentially a process of approximating f (x, y) based on the sampled points matrix Z.

The classical stochastic random field model uses the most general system of parameter characterization, i.e., a probability density function to characterize the height distribution and an areal autocorrelation function to characterize the spatial distribution.

More specifically, for a height matrix Z, the sequence of height distributions z composed of all its elements can be directly characterized by a height probability density function to characterize the surface. However, the probability density functions cannot be well parameterized and are inconvenient to use in engineering. Considering that the probability density functions of most engineering surfaces are in unimodal form, they can be determined by the characterization parameters mean, variance, skewness, and kurtosis, where

(2) $$\begin{array}{c} \mu =\frac{1}{n_{x}ny}\sum_{i=1}^{n_{x}}\sum_{j=1}^{n_{x}}z_{ij}=0,Sq\left(=\sigma^{2}\right)=\frac{1}{n_{x}ny}\sum_{i=1}^{n_{x}}\sum_{j=1}^{n_{x}}z_{ij}^{2}\\ Ssk=\frac{1}{n_{x}nySq^{2}}\sum_{i=1}^{n_{x}}\sum_{j=1}^{n_{x}}z_{ij}^{3},=Sku=\frac{1}{n_{x}n_{y}Sq^{2}}\sum_{i=1}^{n_{x}}\sum_{j=1}^{n_{x}}z_{ij}^{4} \end{array}$$

Here *µ* = 0 is because the preprocessing includes the centering operation. In fact, a more computationally convenient case can be considered in the reconstruction, i.e., the surface height matrix *Z* is further normalized to the case where Sq = 1. This is because the surface height matrix can always be subtracted from the mean of the height series and then divide by the root mean square to achieve standard centrality, *Z_i_* = (*Z-µ*)*/σ*^2^, a process that is reversible. Thus, the surface generated after such processing can be further adjusted to surface that is consistent with the original height parameters.

The spatial distribution of the height matrix, on the other hand, can be characterized by different forms of area autocorrelation functions

(3) $$C_{\tau_{i}\tau_{j}}\left(Z\right)=E\left[Z_{\tau_{i}+x,\tau_{y}+j}\bar{Z_{x,y}}\right]$$

For example the unbiased area autocorrelation function:

$$C_{\tau_{i}\tau_{j}}^{nb}\left(Z\right)=\frac{\sum_{k=0}^{n_{x}-1-i}\sum_{l=0}^{n_{x}-1-j}z\left(k,l\right)z\left(k+i,l+j\right)}{S_{q}\left(n_{x}-i\right)\left(n_{y}-j\right)},\;\{{}_{j=0,1,\cdot \cdot \cdot n_{x}-1_{i}\tau_{i}=i\delta y}^{i=0,1,\cdot \cdot \cdot n_{x}-1_{i}\tau_{i}=i\delta x}$$

the biased area autocorrelation function:

$$C_{r_{i}{,}_{j}}^{b}\left(Z\right)=\frac{\sum_{k=0}^{n_{x}-1-i}\sum_{l=0}^{n_{x}-1-j}z\left(k,l\right)z\left(k+i,l+j\right)}{S_{q}n_{x}n_{y}},\;\{{}_{j=0,1,\cdot \cdot \cdot n_{x}-1_{i}\tau_{i}=i\delta y}^{i=0,1,\cdot \cdot \cdot n_{x}-1_{i}\tau_{i}=i\delta x}$$

and the circular autocorrelation function:

$$C_{r_{i,j,}}\left(Z\right)=\frac{\sum_{k=0}^{n_{x}-1-i}\sum_{l=0}^{n_{x}-1-j}z\left(k,l\right)z\left(k+i,l+j\right)}{S_{q}n_{x}n_{y}},\;\{{}_{j=0,1,\cdot \cdot \cdot n_{x}-1_{i}\tau_{i}=i\delta y}^{i=0,1,\cdot \cdot \cdot n_{x}-1_{i}\tau_{i}=i\delta x}$$

where

$$\{{}_{1+j=1+j-n_{y},1+j\geq n_{y}}^{K+i=K+i-n_{x},k+i\geq n_{x}}$$

As pointed out by Wu [16], the circular autocorrelation function cannot distinguish between correlations with lag distance i δx and (n-i) δx, in the as reflectedimage as symmetric about the midpoint. In contrast, although the circular autocorrelation function cannot distinguish between congruent lag distances, the method is more statistically significant than biased or unbiased autocorrelation from a statistical average point of view. The model can be directly calculated from the circular convolution accelerated by the FFT method, which is very convenient from the computational point of view. In addition, the surfaces generated by arbitrarily specified reconstruction parameters are meaningless for production practice. By performing machining experiments to obtain the relationship between machining parameters and topographic parameters (height distribution and autocorrelation function) and then reconstructing them, the resulting surfaces can be better used as surrogate data for numerical simulation analysis. In particular, it is worth mentioning that when specifying the autocorrelation function, many scholars will give it in the form of the classical exponential autocorrelation function. In fact, it is necessary to give the form of spatial autocorrelation function with processing characteristics according to the specific processing method. For example, for (ultrasonic) grinding surfaces, the simple exponential decay form may not be applicable

It should be recognized that the characterization using stationary random field theory has some limitations. In terms of height distribution, for some special engineering surfaces, such as the surface processed in two processes may have a bimodal distribution, which cannot be well characterized by the moments of the height distribution alone; in terms of spatial distribution, Patir proposed the exponential autocorrelation function for the surface roughness, i.e., the high-frequency component of the surface topography. The autocorrelation function form will lead to the loss of low frequency components for many typical engineering surfaces

### A generic model for topographic reconstruction

Parametric reconstruction is actually the process of solving the optimization problem with the characterization parameters as constraints. In the stationary random field model, typical reconstruction methods include linear filtering model and fast Fourier transform model.

In the case of reconstructing Gaussian surfaces, both Patir's linear transformation method and Hu's FFT method based on finite pulse filtering are computationally similar in nature:

1. Assume that the target surface discrete height matrix *Z* can be obtained from the filter kernel *K* acting on the matrix *R* composed of random sequences
  (4) Z=K⊗R

Where ⊗ is the autocorrelation operator.

2. Determining the target autocorrelation function *C* based on some way, such as calculated from measured surface data or specified in the form of an ideal empirical autocorrelation function.
3. Generating random numbers using SIMD-oriented Fast Mersenne Twister, and then transformed into Gaussian random numbers matrix *R* based on ziggurat algorithm.

**Algorithm 1:** Gaussian surface simulation.

**Input:** target autocorrelation function C	
**Output:** target surface discrete height matrix Z	
1	Generating random numbers using SIMD-oriented Fast Mersenne Twister, and then transformed into Gaussian random numbers matrix R based on ziggurat algorithm
2	Solving the constrained objective function and get the filter kernel K
3	Generating target surface discrete height matrix Z via Equ.(4)
4. Bringing the model assumptions Eq. (4) into the autocorrelation function definition Eq. (3), the constrained objective function is obtained
  (5) C=K⊗K
5. Computing the filter kernel *K* via Eq. (5), bringing it into the original MA model (4), we obtain the surface topography Z that matches the target parameters.

For the reconstruction problem of non-Gaussian surfaces. Steps 3 and 5 need to be further revised.

3-2. The Johnson curve type to which the probability density function of the specified non-Gaussian random sequence belongs and the corresponding parameters can be obtained according to Hill's algorithm, and then the Gaussian random numbers are transformed into non-Gaussian random numbers matrix *R* according to the Jonson transformation.

5-2. The statistical moments of the non-Gaussian random numbers change after filtering. The matrix obtained in the original step 5 cannot satisfy the height distribution parameters. This problem can be solved in the following steps

a. Adjusting the power spectrum density
  $$Z_{\text{new}\;}^{\text{acf}\;}=Z^{h}\frac{\left|Z^{\text{acf}\;}\right|}{\left|Z^{h}\right|}$$
b. Adjusting the height sequence arrangement
  $$Z_{\text{new}\;}^{h}=\text{sort}\left(Z_{\text{new}\;}^{\text{acf}\;},Z^{h}\right)$$

where sort() operation here is to apply the size sorting pattern of $Z_{\text{new}}^{\text{acf}}$ to $Z^{h}$.

When the autocorrelation function is a circular convolution, the solution of the Eq. (5) becomes very simple. Using the properties of the correlation operation, the calculation can be transferred to the frequency domain, i.e.

(6) $$K=\text{ifft}2\left(\sqrt{\text{ftt}2\left(C\right)}\right)$$

However, when the autocorrelation function takes linear autocorrelation, including biased and unbiased autocorrelation, the solution of the Eq. (5) becomes very complicated. Linear correlation needs to be zero complementary in order to be transformed into cyclic correlation, and the inverse operation cannot guarantee the part where the original is zero. In this case, the current studies have been carried out by solving the nonlinear equations, the related nonlinear calculation methods can be found in [43] and are beyond the scope of this paper.

## Recent research progress

Although there have been several reviews on engineering surface modeling, such as Pawlus have published literatures review summarizing around random surface topography modeling [44], surface texture of plateau-honed cylinder liner [45,46]. There have been many new advances in the last two years.

### Parametric characterization aspects

Nayak's seminal work[3], combined with the ideas proposed by Greenwood [2], leads to analytical results via the so-called spectral moment method [47,48] for the summit height and curvature distributions of anisotropic Gaussian topography, which has propelled the development of asperity-based Gaussian rough surface contact analysis models including single-asperity [49] and multi-asperity [50]. That is, the spectral moments *m*_0_, *m*_2_ and *m*_4_ are defined as

(7) $$m_{0}=\text{mean}\left(z^{2}\right),m_{2}=\text{mean}\left({\left(\frac{\partial z}{\partial x}\right)}^{2}\right),m_{4}=\text{mean}\left({\left(\frac{\partial^{2}z}{\partial x^{2}}\right)}^{2}\right)$$

where mean() denotes the arithmetic mean and *z* denotes the section profile height vector in any direction along the surface. The asperity-peak density *η*, the radius of curvature *ρ*, and the standard deviation of asperity-peak height *σ_S_*, are then approximated as

(8) $$\eta =\left(\frac{m_{4}}{m_{2}}\right)\frac{1}{6\pi \sqrt{3}},\rho =0.375{\left(\frac{\pi }{m_{4}}\right)}^{\frac{1}{2}},\sigma_{S}={\left(1-\frac{0.8968}{\alpha }\right)}^{\frac{1}{2}}m_{0}^{\frac{1}{2}}$$

here *α* = *m*_0_*m*_4_*/m*^2^ is the bandwidth parameter.

Sabino analyzed the effect of non-Gaussianity on the statistical geometry of an isotropic self-affine rough surface [51] by comparing the summits height distribution, expected mean curvature and summits density of the rough surface under Weibull probability distribution with Nayak's Gaussian case theory. Regrettably, only the influencing factors and the corresponding trends are reported, and no quantitative analytical expressions are presented for the calculation of asperity-based statistical model [50].

Zhao [52] investigates the evolution of wear characteristics of Gaussian and non-Gaussian rough surfaces based on a non-Gaussian surface reconstruction technique and a hybrid elastohydrodynamic lubrication sliding wear model, and discusses the effects of surface skewness and kurtosis on lubrication and wear characteristics. The results show that as the wear cycle increases, the contact area and contact loading rate of the rough surface decrease before gradually stabilizing, while the friction coefficient and maximum flash temperature increase and then gradually decrease. The increase in surface skewness and kurtosis increases the probability of wear, leading to an increase in cumulative wear, friction coefficient, and maximum flash temperature.

Hansen [53] proposed a new updated framework for the film parameter, Λ*^∗^*, which takes into account the effect of elastic fluid lubrication (EHL) caused by surface irregularities on the microscopic scale (micro-EHL). In this model,

(9) $$\Lambda^{*}=\left(h_{m}+\delta_{a}\right)/z_{0},$$

where the minimum film thickness *h_m_* = ∆*h* + *z_d_*, ∆*h* is the surface distance, *z_d_* is the deformed asperity height. It solved the error problem of classical parameters Λ when the surface consists of long wavelengths.

In addition to asperity-based models, boundary elements and finite elements are also valuable tools for contact analysis. These two methods do not need to deal with the asperity problem and can analyze the height matrix directly.

However, the sampling areas of four surfaces with different surface height ranges were analyzed [54] in achieving comparability and repeatability of area texture measurements, and it was found that part of the morphological parameters are sensitive to the sampling areas. Considering the measured height matrix is always a subset of that obtained by sampling the true topography, Wang [55] uses harmonic interpolation to propose a possible harmonic description for the shape between measurement points and investigates the effect of resolution on the contact characteristics under normal loading and combined normal and tangential loading. This study provides a method for finite element analysis of rough surface contacts that can bring simulation results closer to reality. A non-uniform B-spline global surface interpolation method [56] is used to assist in the construction of a three-dimensional surface that maximizes the maintenance of realistic shapes, and the contact heat transfer problem between rough surfaces is analyzed. Distinct from multi-scale interpolation models, these interpolation methods focus on low frequency features and are more applicable to textured surfaces with processing signatures.

In addition to the initial contact geometry, issues such as multiple loads and running-in process are also of interest for in-service performance analysis. Zhao [57] analyzes the contact stiffness of the loaded surfaces considering the fact that the machined surfaces of the assembly process usually need to be loaded several times. He found that the conduct contact analysis based on pure elastic deformation, which can obtain acceptable and more accurate contact stiffness results. Wang [58] developed a numerical model of rough surface contact with high computational accuracy for spur gears and applied it to wear prediction. Using this model, gear wear was found to decrease with increasing module and pressure angle, but increase with increasing transmission ratio and input torque. These results suggest that reasonable parameter matching is beneficial to improve the wear resistance of gears. The analysis ideas in this article can be referred to in the analysis of other components.

Based on these analysis methods, inverse reconstruction of engineering surfaces can provide efficient numerical analysis inputs for contact service performance of mechanical components [59,60,61].

For details on the rough surface contact model, see [62,63].

### Parametric reconstruction aspects

For engineering surfaces, the process signature surface corresponds to both the manufacturing process and has an influential relationship with service performance, so the reconstruction of process signature surfaces is receiving increasing attention.

The biggest difference between such surfaces and traditional random surfaces is that they have some kind of low-frequency quasi-random component, which is reflected in the autocorrelation function with significant oscillations in the large lag distance part. Simulation methods based on machining processes [64,65] can produce process signature surfaces, and such methods have significant results in optimizing machining parameters. However, there are undeniably some differences between this type of forward simulation and inverse reconstruction. The most direct point is that forward simulation cannot generate the corresponding surface based on the surface parameters.

To solve the problem of inverse reconstruction of the signature surface obtained by ultrasonic vibration-assisted grinding, Li [66] gives an empirical formulation of its characteristic autocorrelation function by forward simulation, and then gives its inverse reconstruction model [67] based on the FFT method. For surfaces containing components with more significant periodicity, Liu [68] generates the statistical parameters of ideal periodic surfaces and random surfaces by decomposing the statistical parameters of the target surfaces, respectively, and then generates surfaces based on the surface parameters and performs synthesis. It solved the problem that conventional filtering methods cannot well achieve the reconstruction of rough surfaces with waviness or large periodicity. Recently, a new method for generating non-Gaussian sequences with specified height roughness parameters has been proposed for processing signature surfaces by shot-peening [69]. In addition, a new shot peening surface autocorrelation function is proposed to accurately describe the reconstructed surface texture.

**Fig. 1:**
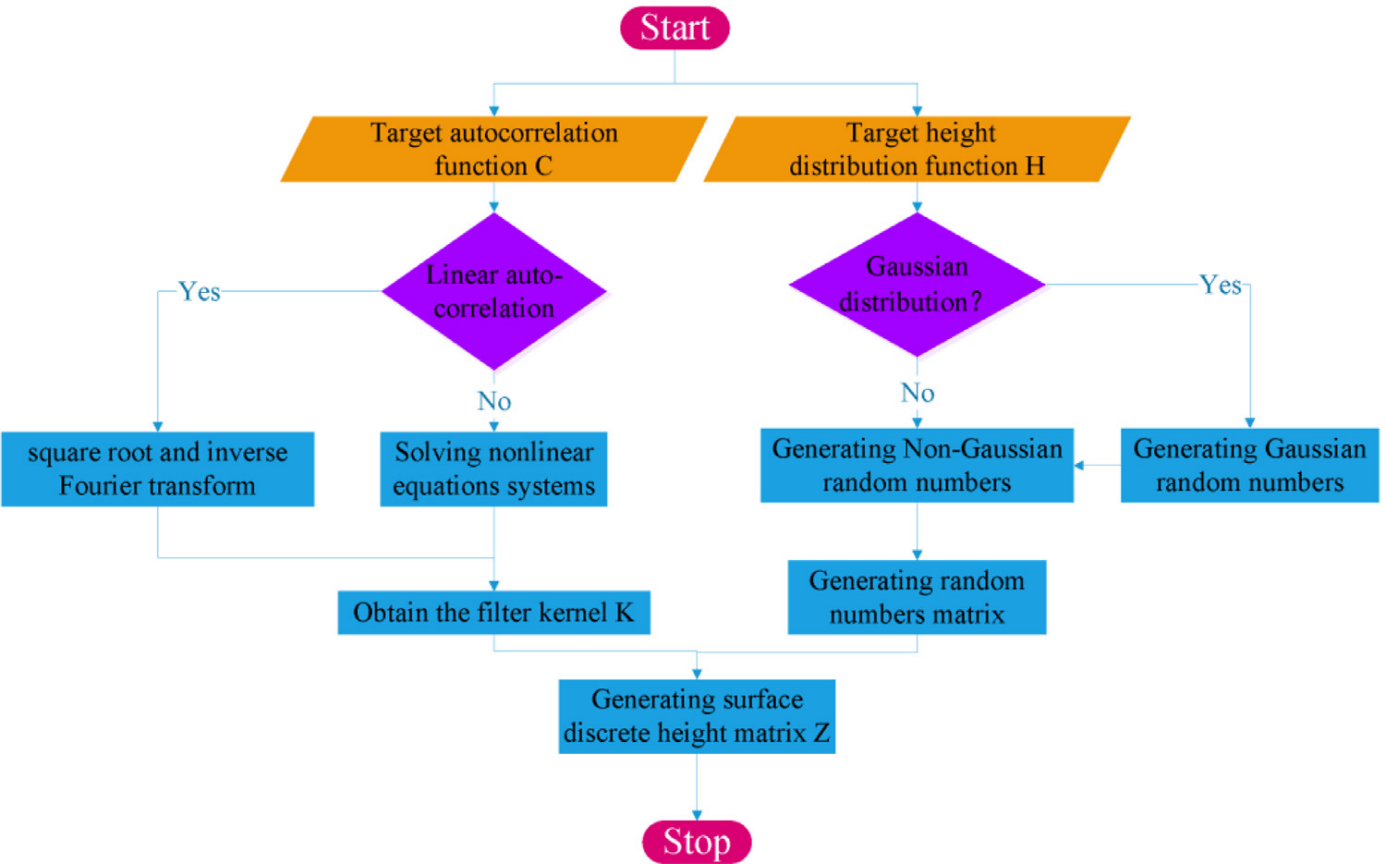
flow chart of reconstruction method.

During the recent years, machine learning has been heavily utilized in engineering. The number of parameters characterizing surface topography is enormous, and for specific performance analysis, inverse reconstruction, and other problems, different subsets of parameters play a major role. All these problems can be analyzed by introducing relevant machine learning algorithms. A combination of statistical theory and data-driven analysis is proposed to solve the problem of characterization of roughness parameters and inversion of grinding parameters when the surface roughness is limited by grinding parameters [70]. Then, by introducing the genetic algorithm and BP neural network, the numerical reconstruction of the grinding surface with the specified parameter set is achieved [71], which solves the problem that the traditional rough surface modeling method based on the stochastic process theory can only associate nine roughness parameters. This research direction has good results in solving some more specific engineering problems and is believed to be a very promising idea. Fig. 1.

**Algorithm 2:** Non-Gaussian surface simulation.

**Input:** target autocorrelation function C and target height distribution function H	
**Output:** target surface discrete height matrix Z	
1	Generating random numbers using SIMD-oriented Fast Mersenne Twister, and then transformed into Gaussian random numbers based on ziggurat algorithm
2	Solving the constrained objective function and get the filter kernel K
3	Transforming the Gaussian random numbers into non-Gaussian random numbers matrix R according to the Jonson transformation
4	Generating surface discrete height matrix $Z^{acf}$ via Equ.(4)
5	Adjusting the power spectrum density and the height sequence arrangement to obtain the target surface discrete height matrix Z

## Conclusion

The dominant representational reconstruction models are now relatively mature. In terms of characterization, there is a transition from typical geometric parameters to performance characterization parameters. The research hotspots have shifted from general rough surfaces to some surfaces with special properties, such as processing signature surface, layered surface superposition, bi-Gaussian surfaces, and directional fractal surfaces. In terms of reconstruction, the basic random field model and FFT method are still the main models for reconstruction. Most of the scholars focus on model solution optimization, such as improvement of nonlinear system of equations solution methods, and model fine-tuning to achieve accuracy improvement. Similarly, with the change of research hotspots in characterization, the corresponding reconstruction models for some special structural surfaces (e.g., wear surfaces) have also received different degrees of attention.

At present, there are still the following fundamental problems in the numerical simulation of surface topography that require careful attention.

• How to solve the accuracy problem of the autocorrelation function in the large lag distance part?

The description of the large lag distance autocorrelation function is necessary to determine the low frequency part of the topographic feature, which plays a key role in the analysis of the adhesive contact problem, while the classical form of empirical autocorrelation function, regardless of the calculation method, is bound to have errors with the engineering “processe signature” surface, which leads to some uncertainties between the design analysis and the manufacturing process.

This also births a subproblem, the scope of application of linear and cyclic autocorrelation function. Are there different effects and thus different ranges of applicability for natural surfaces, engineered “processe signature” surfaces, or is linear autocorrelation always more accurate in terms of characterization?

• The question of whether the bijection is constituted between the reconstruction feature parameters and the contact feature parameters. Note that He [72] and Wang [73] have given a corresponding analysis based on the reconstruction model of FFT method, but there is a curious problem. If the error between the autocorrelation function of the reconstructed surface and the specified autocorrelation function is within a controlled range, then according to the theory, their spatial features should have been similar and the corresponding contact feature parameters should be the consistent, and there should be no difference between different reconstruction method.
• How to reduce the filter kernel size, which in turn makes it more efficient to build convolutional neural network models to reconstruct the processing signature surface.

Current reconstruction frameworks can handle a relatively limited form of autocorrelation functions, and it is an interesting question to draw on solutions that use image processing to solve engineering surface reconstruction problems. However, based on the experience of linear filtering models, the filter kernel size is generally much larger than the case of classical image reconstruction, which will be the main difficulty in this area.

## Ethics statements

The article does not contain any studies with human participants or animals performed by any of the authors.

None

## CRediT authorship contribution statement

**Junye Ma:** Writing – original draft, Writing – review & editing. **Lin Li:** Conceptualization, Writing – review & editing, Funding acquisition.

## Declaration of Competing Interests

The authors declare that they have no known competing financial interests or personal relationships that could have appeared to influence the work reported in this paper.

## Data Availability

No data was used for the research described in the article.
